# The Roman Houses of the Caelian Hill (Rome, Italy): Multitemporal Evaluation of Biodeterioration Patterns

**DOI:** 10.3390/microorganisms11071770

**Published:** 2023-07-06

**Authors:** Daniela Isola, Flavia Bartoli, Simona Morretta, Giulia Caneva

**Affiliations:** 1 Department of Sciences, University Roma Tre, Viale Marconi 446, 00146 Rome, Italy or flavia.bartoli@cnr.it (F.B.); giulia.caneva@uniroma3.it (G.C.); 2 Department of Economics, Engineering, Society and Business Organization (DEIM), University of Tuscia, Largo dell’Università snc, 01100 Viterbo, Italy; 3 National Research Center (CNR), Institute of Heritage Science, SP35d, 9, 00010 Montelibretti, Italy; 4 Soprintendenza Speciale di Roma Archeologia Belle Arti Paesaggio, Ministero della Cultura, Piazza dei Cinquecento, 67, 00185 Rome, Italy; simona.morretta@cultura.gov.it; 5 National Biodiversity Future Center (NBFC), Università di Palermo, Piazza Marina 61, 90133 Palermo, Italy

**Keywords:** carbonatogenic bacterial activity, cyanobacteria, *Cyphellophora olivacea*, fungi, lampenflora, oligotrophic environment biodiversity, *Scytonema*, soil actinobacteria, wall-painting conservation, underground cultural heritage

## Abstract

Like other hypogeal environments, the Roman Houses of the Caelian Hill are prone to unwanted biological growth. Wide conservative interventions have been carried out at the beginning of this millenium to reduce biodeterioration and physical–chemical damages. Retracing the last monitoring work, we assessed the site’s current state of conservation and biodeterioration intending to check the previous treatments’ effectiveness and deepen the common knowledge of the subterranean biota and their possible biodeteriogenic effects. Starting from the past test areas and the previous identifications of the occurring biodeteriogens, we further isolated and identified the main eubacterial, fungal, and phototrophic settlers, focusing on some detrimental traits for wall paintings (i.e., acid production and carbonate precipitation). The achieved results proved the success of the performed interventions in reducing the wall’s water content. Otherwise, the new conditions raise, in the long term, new concerns about lampenflora, carbonate precipitations, and salt efflorescence. Here, the Caelian Houses’ new status is documented. The possible favouring conditions for the different groups of biodeteriogens, along with the taxonomical novelties, additional risks tied to the anthropization of the resident culturable microbial community, and the possible relation between the black fungus *Cyphellophora olivacea* and roots, are reported and discussed.

## 1. Introduction

Hypogeal sites are, in general, nutrient-poor ecosystems characterized by very high air relative humidity, sometimes close to saturation, high water content in the wall structures, constant relatively low temperatures, and poor ventilation, which make them particularly favourable for a wide number of organisms [1]. Moreover, underground environments are considered “extreme.” As for Liebig’s law of the minimum, in this environment, the main limiting factors are the light for photoautotrophs (for lichens also ventilation) and the availability of organic matter for heterotrophs; for this reason, common inhabitants of these sites are phototrophs adapted to the scarcity of light and oligotrophs adapted to long periods of starvation [2,3,4,5]. The environmental pressure expressed by limiting factors is so high that each small change determines rapid variation in the resident biotic communities, producing different visible biodeterioration patterns (BP) [6]. Changes in light, carbon dioxide, nutrient inputs, and temperature have been recognized as major driving factors for sudden changes in underground microbial communities [2,7,8]. Allochthonous carbon energy sources can be tied to outdoor airborne particles, arthropods, roots, and materials used in wall paintings [2,9,10,11,12] or frequently to mass tourism [13,14,15]. Indeed, human presence can affect microclimatic conditions since it increases the indoor carbon dioxide, temperature, and humidity content [16,17,18]; otherwise, the visitors’ shoes and clothes are vehicles for organic and inorganic outer particles [1,5,19,20].

These conservation problems of hypogeal sites are widely known, and general suggestions for their maintenance is often reported. It has been observed that along the visitor trails the growth is higher than others [13,16,19,21]. The most common actions aimed to limit the access/flow of visitors and their permanence in the site, and, in extreme cases, provide the use of protecting clothing to avoid the input of outdoor contaminants and the changing of environmental condition [2,22,23,24]. Many attempts have been performed to maintain under control the lampenflora growth, using, for instance, the installation of monochromatic lights with limited wavelength emission [25,26] or tunable low-energy-consuming LED lamps with the emission spectra lying out of the absorption maxima of chlorophyll a [27,28,29,30]; moreover, this indirect control measure may fail in the long term [7].

The biodeterioration problems of Roman hypogea have been extensively studied for over 40 years. Different species of terrestrial epilithic cyanobacteria were detected as dominant phototrophic microorganisms [31,32,33] followed by bacteria and, in a lesser extent, by green algae and fungi [2,34,35,36]; mosses and roots of higher plants were also present [22,23,37,38,39]. In Roman hypogea, different biofilms whose composition changes according to microclimatic conditions and lamps are evident [33,35,40,41,41,42,43,44,45,46,47]. In fact, when light is available, a massive growth of cyanobacterial-dominated microbiota has been reported [25]. Otherwise, in the darkness, white biofilms of different consistency and origin were found [16,25,47,48]. In all cases, a high diversity of heterotrophic microflora was recorded, and several new bacterial species were described [49,50,51,52,53,54], confirming that the unexplored and/or extreme niches are reservoirs for unknown entities [5,55,56,57,58].

In cultural heritage conservation, the importance of long-term studies lies in measuring and documenting nature’s response to the changes imposed by treatments as previously done, for example, for the Matera’s paintings in a rocky habitat [59,60] and, meanwhile, allowing preventive actions when and if the first signs of a dangerous situation are recognized. In this light, the Roman Houses of the Caelian hill represent an interesting case since broad conservative interventions to reduce biodeterioration, and physical–chemical damages were carried out in the first years of such millennium before opening to visitors [22,23]. Hence, using the past data in this paper, we aim to: (a) analyze the current conservative conditions after about two decades from the first interventions; (b) compare the results of the previous monitoring work with the present state of conservation and biodeterioration of the site; and (c) assess the detrimental potential of the occurring species. This diachronic study will allow to establish who (referred to the microbial components), why, and what has changed. It will also allow for the identification of the potentially deteriogenic species and the improvement of the management strategies for the protection of the site.

## 2. Materials and Methods

### 2.1. Study Site, Previous Intervention, and Analyses

**Figure 1:**
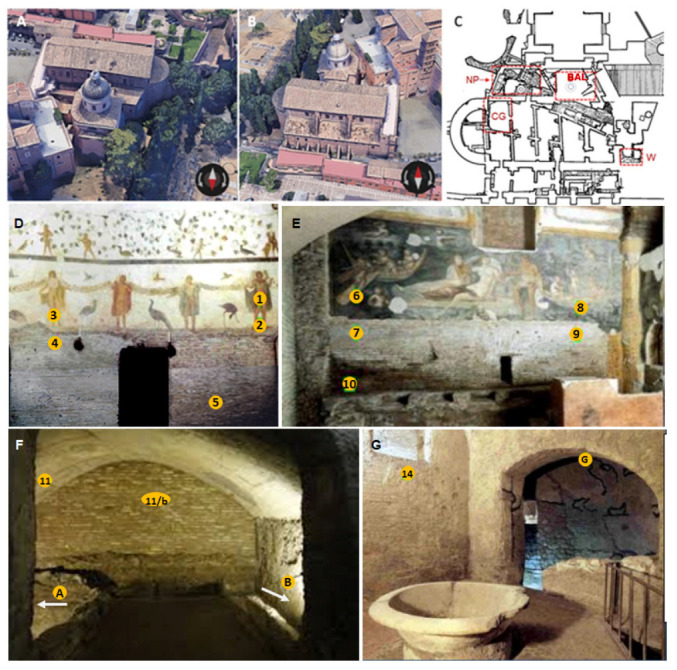
Roman houses of the Caelian Hill. (**A**) Aerial view, Northern side, (**B**) Aerial view, Southern side; (**C**) Site map, in red, are the four rooms under study and after the pictures of them as they were during the early 2000s: (**D**) Chamber of Geniuses (CG), (**E**) *Nymphaeum* of Proserpine (NP), (**F**) The Winery, (W), and (**G**) The “*Balneum*” the white arrows indicate the A and B walls where sampling was performed (BAL). Within yellow are the sampling points used by Bartolini and colleagues [22,23].

Roman houses, object of this study, are located between the Coliseum and the Circus Maximus in the heart of ancient Rome (41°53′11.04″ N 12°29′31.2″ E). In the first century B.C., high *insula* buildings flanked the *Clivus Scauri*, the western main route of the Caelian hill (*Collis Caelius*). Here, arcades, shops, and entrance doors of apartments on the upper floor made this street very busy and full of activities, which continued until the third century [61]. Many building transformations occurred to meet the needs of the population and owners during the time until the second half of the fourth century when it became a place of worship. The most striking example of this transformation is the series of bricked up windows that became part of the Basilica dei Santi Giovanni e Paolo (Basilica of Saints John and Paul), which is visible even from the outside (Figure 1A,B). This inclusion in the new structure changed drastically the houses space ratios and the lines of floors overlapping and caching the pre-existing structures, transforming *de facto* houses into a false hypogeum. The *insula* was used until the 9th century, then abandoned and discovered at the end of the 19th century. In 1997, the Soprintendenza Speciale per i Beni Archeologici di Roma (Special Archeological Superintendence of Rome), with the collaboration with the Central Institute of Restauration (ICR), started the restoration of the Roman houses facing several problems of paintings conservation due mainly to water infiltration and hypogeal microclimatic conditions.

Before restoration and opening to visits (July 2002) the ICR performed a study along a two years’ time lapse, to understand the biodeterioration dynamics, support a preventive measures plan, and schedule a prevention timetable [22,23]. With this purpose, a quali-quantitative investigation has been performed on surfaces’ contamination by heterotrophic and autotrophic microorganisms. Four rooms along the visit path were used as a reference for the environmental conditions characterizing the site. In detail, the “Stanza dei Geni” (the Chamber of the Geniuses, CG, the household spirits protecting home) is close to the entrance, the “Ninfeo di Proserpina” (the *Nymphaeum* of Proserpine, NP) is a large room with the external wall partially buried and in contact with the external garden and by air with the space downstairs. More, the “cella vinaria” (the winery, W), a three-sides closed cell characterized by highly moist walls, and the “*Balneum*” (a private thermal plant, BAL) in the deepest floor, never treated or restored and excluded from the visit path (Figure 1). The paintings decorating the CG and NP were prepared using the fresco technique with a whitish and blue dominant background colour respectively.

### 2.2. Present Investigations: Sampling, Environmental Measurements and Biodeteriogens Identification

#### 2.2.1. Field Analysis: Microclimatic Measurements and Sampling

To obtain consistent and comparable data, we identified the previously analysed areas [22,23] and applied the same sampling techniques and types of qualitative-quantitative analyses. On 19 July and 15 November 2019 two sampling campaigns have been performed. The date choice was subject to sampling permissions, the absence of open visits to the public and the four-month gap to verify the presence of thermal inversion.

Using sterile swabs, 16 samples were taken from 9 cm per side squared areas defined by plastic sheets (one for each sampling point) as performed by Bartolini and colleagues in the previous studies ([22,23]; Figure 1D–G). Additional samples were taken to investigate the areas showing signs of biological growth such as discolorations or areas at risk for biological growth (e.g., illuminated areas), adhesive tape sampling method was also used. Dried tiny roots were recorded in the NP below the fresco in the main wall (right side) and sterilely collected. Samples taken from green patinas were stored at 4 °C and processed within the following two days; the other samples instead were stored at −20 °C and processed immediately after. 

During sampling, the temperature (°C) and relative humidity (RH%) measurements were made using portable thermo-hygrometer Extech MO290 (Extech Instruments, Nashua, NH, USA). 

#### 2.2.2. Cultivation, Isolation, and Identification of the BP’ Components

The samples were suspended in sterile saline (NaCl 0.9%), diluted scalarly and then plated in triplicate on Mycological agar (MYC, BD Difco^TM^ Sparks, MD, USA) consisting of soy peptone 10 g/L, dextrose 10 g/L, agar 15 g/L and incubated at 28 °C following UNI NORMAL 9/88 protocol to count the total number of fungi and bacteria expressed as CFU/cm^2^ [22,23,62].

Fresh material was observed in a Thoma chamber where frequency values were achieved for phototrophic taxa. An aliquot of 100 µL of sample suspension was inoculated into liquid BG11 freshwater medium (BG11, Sigma Aldrich, Darmstadt, Germany) and Bold Basal liquid Medium (BBM, Sigma Aldrich) developed for cyanobacteria and micro-algae respectively. Cultures were maintained 6–8 weeks under cool-white fluorescent illumination (Osram Dulux L 36W/840 Lumilux, 2900 lumens, Osram GmbH, Munich, Germany), with a 12-h photoperiod, at 20 ± 2 °C [22,23,63]. Once plated onto a solid medium, was left to grow at the abovementioned conditions until colonies were detectable and picked up for molecular identification not performed in the previous survey.

To improve the isolation yields and knowledge on current microbial community, 100 μL of each serial dilution (from all the samples taken) were plated also on Luria-Bertani (LB) for bacteria, Starch Casein Agar (SCA) for actinomycetes, Trypticase Soy Agar (TSA) supplemented with NaCl (3% *w*/*v*) and MgSO4·7H_2_O (2% *w*/*v*) for salt tolerant bacteria, Dichloran Rose Bengal Chloramphenicol (DRBC, VWR International GmbH, Darmstadt, Germany) for fungi. Plates were incubated for two months at 20 ± 1 °C for bacteria and 15 ± 1 °C for fungi to be more consistent with site conditions and improve the detection of slow-growing microorganisms.

The isolates in pure cultures were transferred on tryptic soy agar (TSA) for bacteria and malt agar (MA, Malt extract 30 g/L, bacteriological agar 15 g/L) for fungi and selected for further processing based on major morphological colony features.

Molecular identification of bacteria was performed using 16S as molecular marker and 27F/1492R as primer set (5′-AGAGTTTGATCMTGGCTCAG-3′ and 5′-GGYTACCTTGTTACGACTT-3′ respectively). Specific primer pair Cya106f (5′-CGGACGGGTGAGTAACGCGTGA-3′) and Cya781r (5′-GACTACWGGGGTATCTAATCCCWTT-3′; [64] for cyanobacteria were also used. The nuclear internal transcribed spacer (ITS) flanked by ITS4/ITS5 primers (5′-TCCTCCGCTTATTGATATGC-3′/5′-GGAAGTAAAAGTCGTAACAAGG-3′; [65]) was used for fungi and root sample identification. While primer sets targeted to chloroplasts such as ChloroF/ChloroR (5′-TGGCCTATCTTGTTGGTCTGT-3′ and 5′-GAATCAACCTGACAAGGCAAC-3′ respectively; [66]) and rbcLa/rbcLr590 (5′-ATGTCACCACAAACAGAGACTAAAGC-3′ and 5′-AGTCCACCGCGTAGACATTCAT-3′; [67]) were used for the molecular identification of green algae and roots respectively. 

PCR reactions were performed in a total volume of 25 μL using BioMix (BioLine, Luckenwalde, Germany), 5 pmol of each primer and about 30 ng of template DNA were added. Amplifications were carried out using MyCycler™ Thermal Cycler (Bio-Rad Laboratories, Munich, Germany), the protocols used are listed at Table S1. Sequencing was performed by Macrogen (Madrid, Spain) and the electropherograms manually checked/assembled using Chromas Pro 1.41 (Technelysium, Southport, QLD, Australia). Similarity searches have been performed using the algorithm BLASTn limiting the search between the sequences coming from type strains for bacteria and excluding from the comparison “uncultured/environmental sample sequences” for fungi. The bacterial taxonomical ranking was fixed using NCBI taxonomy browser as reference. The obtained sequences were deposited in GenBank. Morphological identification of phototrophs was performed using the analytic keys of Guiry and Guiry [68].

### 2.3. Multitemporal Analysis

According to Bartolini and colleagues [22,23], the presence of phototrophic and heterotrophic microorganisms has been assessed, the latter discriminating between fungi and bacteria. To visually describe the variation that occurred over time in the communities analyzed, a quali-quantitative scale of frequencies has been set. In detail, CFU/cm^2^ were reported as values (+) in order of magnitude with the power of ten (i.e., +, ++, and +++ were used instead of values 10, 100, and 1000 respectively); while +/− indicate positive values below 9, - not found; and / not investigated.

The availability of morphological identification of the phototrophic component [22,23] allowed the comparison at the species level. Otherwise, a comparison at the higher rank (i.e., as fungi and bacteria) was performed for the heterotrophic microorganisms.

### 2.4. Plate Assay for Bacterial Detrimental Potential

Fungal spreading is a rare event in hypogeal environments generally tied to sudden changes in carbon sources availability or the weakening of the resident bacterial community with antifungal activity due to biocide treatments [1,5,9,14]. Since no active fungal growth was recorded before the opening to visit [22,23] nor recently, we decided to focus on the heterotrophic bacterial component and its adverse potential. Using plate trials, we investigated the acid production and carbonate precipitation possibly leading respectively to substrate dissolution and pigments alteration [69], and interfere with the visual appreciation of the underlying artwork [70]. Acid production was assessed using CaCO_3_ agar medium containing yeast extract 5 g, glucose 50 g, CaCO_3_ 5 g, agarose 15 g per liter, while precipitation test was carried out on B4 medium (yeast extract 1 g, glucose 1 g, calcium acetate monohydrate 5 g, agarose 15 g per liter of solution). Mineral phase precipitation was monitored twice a week under a stereomicroscope (Nikon SMZ80, Minato, Tokyo, Japan) and documented by Nikon Coolpix 500 camera.

## 3. Results

### 3.1. Current Conservative Conditions

From a microclimatic point of view, the performed measurements evidenced as the rooms investigated are subject to a gradient of temperature and relative humidity (Table S2) and as the outdoor conditions significantly affect the Geniuses Chamber (CG). In contrast, stable conditions characterize the *Balneum* (BAL). Indeed, the variation recorded in CG during Summer and Autumn sampling sessions was about 4 °C and 8 RH%, while 0.2 °C and 1 RH% in BAL. Moreover, even in BAL, the RH levels were below 90%.

Comparing the presence of microclimatic conditions to the data extracted in 5 November 2002, we can note that temperatures are superimposable while major RH differences have been recorded up to 7% (Table S2).

**Figure 2:**
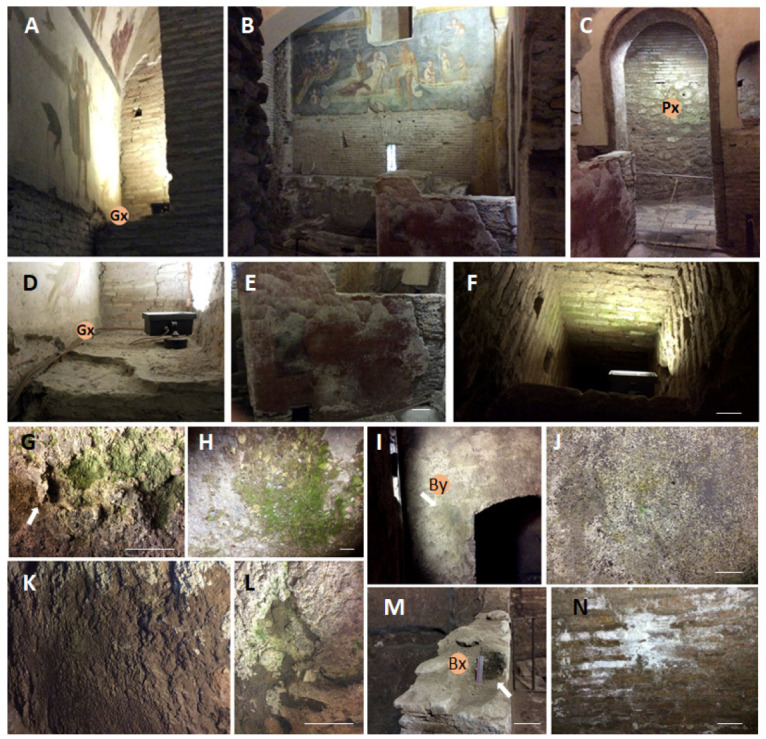
Deterioration patterns recorded and additional sampling points. (**A**,**D**) GC, niche—additional sampling point; (**B**,**E**) NP, lower wall–additional sample; (**C**) Light green opaque patina at the NP side chamber; (**F**) Lampenflora example along the visit path; (**G**,**H**) Winery deterioration pattern recorded on the A wall; the white arrow indicates a exfoliating flake from the wall (**K**,**L**) Winery-deterioration pattern recorded on the B wall characterized by a brown patina in the lower shaded part (**K**) and a green one close to the light (**L**); (**I**,**J**,**M**,**N**) *Balneum*: (**I**,**J**) Illuminated wall, By sampling site, and magnification of the yellow–green discoloration pattern; (**M**) Greenish colonized lower wall from where Bx sample was extracted, (**L**) Whitish patina on the non-irradiated lobby wall. Bar is equal to 10 cm.

The most common biodeterioration pattern resulted green biofilms close to lamps (Figure 2F). Particularly evident in the walls of the winery (Figure 2G,H,L), the green patinas seem to be here associated, in the closest part to the light, with mortar weakness since in wall A it exfoliates (Figure 2G white arrow) while the wall B (Figure 2L) when gently knocked it produced a dull sound.; more in the shaded part of the wall B a brown patina was also visible (Figure 2K). Lampenflora has also been recorded in the side chamber of the NP where the bricks are covered by a powderly opaque light green patina (Figure 2C, Px sampling point) and in the *Balneum* with two new sampling points namely By (Figure 2I) and Bx (Figure 2M). A white patina due to salt efflorescence, not recorded before (Figure 1E), has been found in the lower wall in the NP (Figure 2B,E). 

### 3.2. Identification of Biodeteriogens

**Figure 3:**
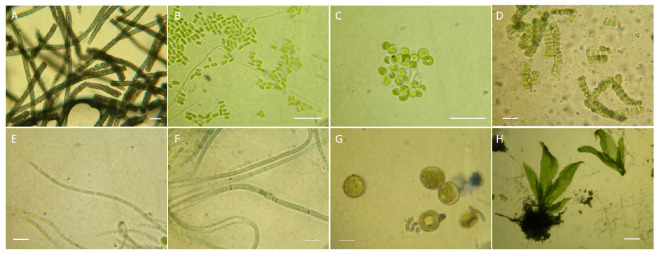
Phototrophs optical microscope images. (**A**) NP, *Scytonema* sp. from sample Px; (**B**) W, wall A—*Pseudostichococcus monallardoides*; (**C**) W, wall B—*Chlorella vulgaris*; (**D**) BAL, *Desmococcus vulgaris* from site G; (**E**) *Albertania* sp.; (**F**) BAL, *Phormidium laminosum* from site B15; (**G**) BAL, *Chlorococcum vulgaris* from site Bx; (**H**) BAL, young moss gamethophytes, Site By. Scale bar 15 μm in (**A**–**G**), 50 µm in (**H**).

The direct observation at microscope allowed for the identification of phototrophs in some cases improved by the molecular analysis. The NP side chamber (Px) showed a light-green opaque patina on the main wall composed exclusively by *Scytonema* sp. (Figure 3A). The Wall A of the winery is dominated by *Pseudostichococcus* cfr. *monalloides* (identity 98.32%; OQ540755; Figure 3B) while *Scytonema* sp. was rarely found in fresh microscope preparation only. Wall B showed the prevalence of *Chlorella vulgaris* while molecular analysis evidenced the presence of *Koliella* sp. (93.04%, OQ540756) from this sampling site. All BAL sampling sites showed a varied presence of primary producers represented by *Albertania* sp. (*A*. *skiophila* 98.68%, OQ534285) and *Coccomyxa* sp. (97.21%, OQ540754) in Site P14, and Leptolyngbiaceae sp. (*A. alaskaensis* KL12 93.18%, OQ534284) in Site P15. The sample extracted from the vault (G) revealed the presence of *Desmococcus vulgaris* (Figure 3D). While Sample By evidenced the huge presence of young leafy gametophytes and mosses rhyzoids (Figure 3H), *Stichococcus* sp. along with *Chlorococcum vulgaris* were found in Site Bx.

One hundred fifty-one bacterial isolates were achieved, while 87 were selected by colony morphology and further processed. The bacterial identification (Table 1) evidenced the genus *Peribacillus* as the most frequent, as it was recorded in three chambers (CG, NP, and BAL) and represented 17.44% of isolates. At a higher rank, the class Bacilli (36.78%), the order Bacillales (36.78%), and family Bacillaceae (32.18%) are the most frequent (Figure S1).

Twenty-six fungal isolates were achieved (Table 2). They mainly belong to the genera *Penicillium* (42.85%), *Cladosporium* (23.8%), and *Aspergillus* (4.76%). The remaining strains (28.57%) are represented by strains of the genera *Malassezia*, *Torula*, *Chrysosporium*, and *Cyphellophora* (*C. olivacea*); the last two had 97.1% identity with *Pseudogymnoascus pannorum* and 96,45% with [*Coniosporium*] MA 4639. *Centaurea, Echinops,* and *Saussurea* resulted in the best matches for *rbcL* root sequencing (100%, OQ550265), while the ITS target led to the amplification of the fungus *Cyphellophora olivacea* (99.3%, OQ534303). *Penicillium* sp. was isolated from PX, CV-A, and CV-B (green patinas). Still, its finding should be considered “occasional” since only one colony per site has been found there, and direct microscope observation of green patinas never recorded the presence of hyphae.

### 3.3. The Multitemporal Evaluation

#### Total Counts Comparison

Comparing the results of the previous monitoring work with the present and biodeterioration of the site, trough plate counts, we can observe that the number of heterotrophs decreased in the samples taken from Sites G5 and P10, which were previously affected by a brown and grey-brown patina respectively (Table 3). A decrease has also been recorded in the winery (Samples 11 and 11b) and *Balneum* (Sample 14). Otherwise, a marked increase on counts has been recorded especially in the Nymphaeum (NP) in Samples P7, P8, and P9. Meanwhile, G1 and G2, which were never investigated before for heterotrophs, revealed a high presence of fungi. An increased presence of phototrophs was found in the winery and in all sampled sites in the *Balneum* (Table 3).

Regarding the phototrophic microorganisms, the comparison with the previous data showed their complete disappearance in P10 samples (Table 4). The Winery’s walls, characterized by an evident green patina, recorded *Pseudostichococcus, Scytonema*, and *Chlorella* species; otherwise, the *Balneum* recorded the disappearance of the main patina from Sample G extracted along the arch (Figure 1G) and the appearance of new ones where even the presence of moss gametophytes have been recorded as in Sample By (Figure 2I,L).

### 3.4. The Assessment of the Detrimental Potential of the Occurring Bacterial Species

**Figure 4:**
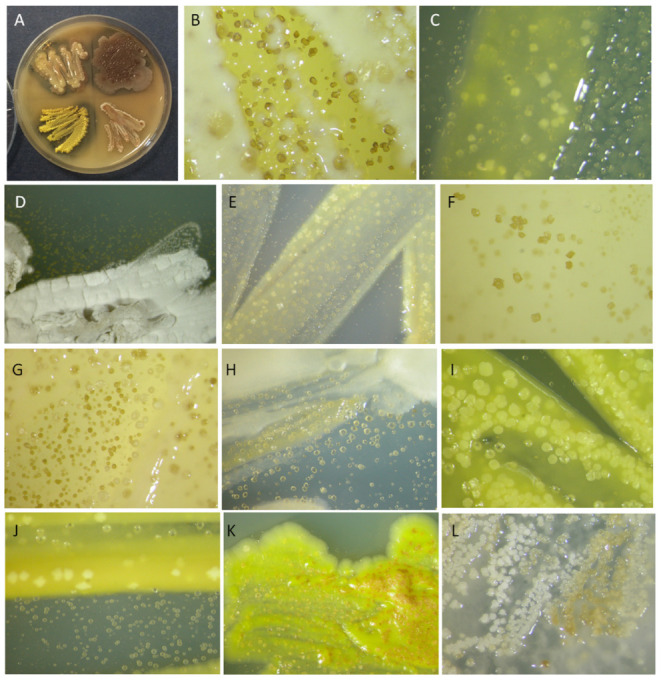
Bacterial isolates detrimental potential. (**A**) Acid production leading to carbonates dissolution; (**B**–**L**) Carbonates precipitation differing in shape and colour. Magnifications from B4 plates of the strains: (**B**) *Lysinibacillus* sp. B35; (**C**) B106; (**D**) *Amycolatopsis* sp. B79; (**E**) Alcaligenaceae sp. B71; (**F**) Bacillaceae sp. B65; (**G**) Bacillaceae sp B66; (**H**) *Stenotrophomonas* cfr. *maltophilia* B105; (**I**) *Microbacterium* sp. B76b; (**J**) *Lysinibacillus* sp. B70; (**K**) *Agromyces* sp. B58; (**L**) *Peribacillus* sp. B61. Magnifications were 4× except (D), whicht was 2×.

The bacterial isolates frequently showed the ability to precipitate carbonates (84.7%) while the ability to dissolve them was recorded in 29.4%. A small portion of them (5.9%) demonstrate none of the deteriorative ability at the tested conditions, while 20% had both. In the tested strains, the carbonatogenic phenomenon varied by quantity (Table 1, indicated by +, ++, +++) and quality (Figure 4) The carbonatogenic phaenomenon varied by quantity and quality in the tested strains (Figure 4, Table 1). Strains of the genera *Achromobacter*, *Alcaligenes*, *Agromyces, Lysinibacillus*, *Nocardia*, *Peribacillus*, and *Stenotrophomonas* were the most active precipitating carbonates. Furthermore, the colour of precipitates ranged from whitish to brown, and their shape was from spherical to crystalline.

## 4. Discussion

Although the crucial importance of long-term surveys is widely recognised for the conservation of cultural heritage [71], only a few researches have been performed in this light. Changes in the conservative priority objectives for site conservation and diagnostic methods used are the primary constraints for this kind of investigation based mainly on data comparison and the ability in deciphering the future trends.

The biodeterioration patterns found in the Roman Houses are consistent with those reported in visited hypogeal sites worldwide [1,72,73] as well as the record of new taxa within the bacterial, fungal, and phototrophic settlers. Such data also confirms the importance of the multi-temporal investigation for checking the effectiveness of the previous treatments [59,60]. The occurred changes appear to be a clear consequence of the direct and indirect control methods applied before opening to visit, but in the meanwhile, this study added new insights into the underground biota. The duration of application of biocides can have a maximum duration of 3–4 years in a favourable condition, such as humid places [74]. The interventions applied to reduce the walls dampness threatening the frescoes conservation are long lasting and effective [4] and it is confirmed here since the total counts notably decreased, and brown biofilm and diatoms disappeared from G5 and P10 in the CG and NP respectively. Diatoms also disappeared from BAL demonstrating as the applied interventions affected even the inners floors. Indeed, if we recently recorded an average RH of 88.1% before it was always above 90% (93% measured 5 November 2002) [22.23]. After that, following the basic Liebig’s law the heterotrophic communities confidently redistributed themselves driven by water gradient and carbon sources availability. For this reason, even the most common distribution of bacteria and fungi on vertical surfaces (namely bacteria/down and fungi/up) deserves a reading up in the light of the species involved and their ecology. Bacteria largely prevailed in number and diversity, showing a dominant carbonates-precipitation trait. This feature, widely reported in the literature [75,76,77,78], could threaten wall painting conservation beyond the interest for biotechnological applications [47,79,80], and needs further knowledge improvements to understand their role in the underground environments better. Meanwhile, colourimetric measurements could be scheduled for the wall paintings to verify possible changes in lightness or colour. While RH environmental monitoring is required to reduce/avoid further saline efflorescence and ensure the best conditions for wall paintings conservation. 

Other detrimental treats deserving attention could be indirectly ascertain by the taxonomical composition of bacterial community. The recorded bacterial community is composed for about one third (35.29%) by Bacilli, 27.06% by Actinomycetes and 35.30% by Alpha-, Beta-, and Gamma- proteobacteria. The recurrent presence of pathogenic or opportunistic species can be considered a mirror of the human impact on the environment [2,55,81], while microorganisms of anthropogenic origin a consequence of increased availability of organic matter introduced by visitors [13]. In this sense, the finding of species of the genera *Inquilinus*, *Amycolatopsis, Nocardia, Nocardioides, Streptomyces,* the family *Micrococcaceae* on the one hand [55] and *Alcaligenes faecalis* and *Staphylococcus capitis*, on the other seems to confirm this trend. Along with the human-impact on the microbial community there is another aspect deserving attention from a conservative point of view: the resistance to biocides and/or antimicrobials. Actinobacteria and Firmicutes (now Bacillota) are able of carrying and disseminating ARGs (Antibiotic resistant genes; [82]) and recently has been assessed that in stone monuments microbiotas Actinobacteria and Proteobacteria averagely accounted for 39.7% and 39.0% respectively of ACCs (Antimicrobial resistance Carrying Contigs; [83]). Moreover, some resident bacterial strains can have antifungal activities like species of *Micromonospora*, *Rhodococcus Streptomyces*, *Bacillus*, *Pseudomonas*, *Stenotrophomonas* [14]. So, in case of direct actions, is of utmost importance to carefully evaluate the biocide chemical features as well as the spectrum of efficacy on the resident community to avoid the selection on resistant bacteria or fungal spreading as reported for Lascaux cave [1,5,84].

This principle is valid also for fungi even if in the Roman Houses the situation recorded do not seem to raise concerns. The isolated strains, according to other subterranean cultural heritage sites, mainly belong to the cosmopolitan highly sporulating genera *Penicillium*, *Cladosporium* and, in lesser extent, *Aspergillus* and their presence is related to the air flow from the entrance and within the site [85,86,87]. More interest raises from the other isolates closest relative of the species *Malassezia restricta* (97.46% identity), a species associated to infections in humans [88], or the presence of “black fungi” relatives to [*Coniosporium*] MA 4639, previously isolated from marble monuments [89] and *Cyphellophora olivacea* a species previously found in subterranean environments and hydrocarbon contaminated sites [90,91]. While, the relative of [*Coniosporium*] MA 4639 was little represented in a site characterized by huge debris, *Cyphellophora olivacea* was dominant in both P9 samples taken at NP [92]. More interestingly this species was also amplified from the root sample taken there (NP). This fact and previous records of its siblings from roots [93] corroborates the hypothesis of endophytism (dark septate endophyte, DSE) and confirms the detrimental role played by roots in the conservation of hypogeal cultural heritage sites [5,11]. The root identification by rbcL sequencing should be confidently ascribed to Asteraceae family because the species recording the best match are not part of the Latium flora [94] and the well-known poor representation of the local flora in GenBank [11]. In any case, roots finding deserves attention and external perimeter walls inspected. 

Aside from the widely reported aesthetical alteration, phototrophs have been associated with stone surface decay. They can cause chemical and physical damage to stone surfaces by producing chelating agents and acids [95]. Calcium carbonate precipitation has often been recorded in association with algal growth in caves and mural paintings threatened by calcite deposition [96]. Most phototrophs grow following the topology of the mineral surface layer, while others contribute to the formation of micro-fissures growing just below it or actively bore the mineral substrata [4,35,97]. This activity has been documented, for example, for *Scytonema*, where calcium carbonate deposition on cyanobacterial filaments has frequently been recorded at subterranean sites [2,33,35,36]. Anyway, the finding of new taxonomical entities within the genera *Albertania*, *Coccomyxa* and *Koliella*, confirms underground sites as reservoir for new species and underline the importance of this kind of investigations [86,90]. Algal and cyanobacterial components were differently space distributed. For example, even if cyanobacteria are the most adaptable phototrophs due to their tolerance to desiccation and low light intensity requirements [98], in habitats where water is available and, in general, characterized by less environmental stress like illuminated spots around lamps, they are quickly overgrown by fast-growing eukaryotic algae [99]. Indeed, algae prevailed in site characterized by wall dampness (close the ground) and directly illuminated as the two walls of the Winery, while cyanobacteria dominate aside lights far from ground (Px). In this light, is obvious that one of the first actions to be done concern the illuminating system with the use of new generation lights (e.g., LED) not closely facing walls and possibly timed. Furthermore, as the *Balneum* was (i) never treated before, (ii) is constantly illuminated even if out of the visit path, (iii) is *in continuum* with the upper floors and (iv) is subject to temperature inversion affecting the air flow, (v) showed a notable increase of the phototrophic patinas (e.g., young gametophytes where they are not) could serve as reservoir for phototrophs spreading. 

## 5. Conclusions

Research strategies on biodeterioration are changing, and ecological studies applied to artwork conservation could represent a compelling tool to improve the intervention durability and apply the principle of “minimum intervention” best. Long-term studies also provide a model for preventive actions based on BPs as bioindicators of warning conditions (e.g., the carbonatogenic trait characterizing our bacterial isolates). 

Environmental monitoring and understanding the ecological successions are essential in preventing site re-colonization through appropriate conservation plans. Still, the knowledge of the involved species and their main traits is superficial.

## Figures and Tables

**Table 1:** Identification and detrimental potential of the bacterial isolates.

Sampling Point	Isolate	Closest BLASTn Match	% Identity	Accession N	CaCO_3_	B4
G1	B59	*Streptomyces pluricolorescens* NBCR12808	99.62	OQ533438	−	−
	B82	*Peribacillus simplex* NBRC 15720 = DSM 1321 *Peribacillus frigoritolerans* DSM 8801 *Peribacillus castriliensis* N3	99.10	OQ606394	−	++
G2	B103	*Phyllobacterium zundukense* Tri-48 *Phyllobacterium loti* S658 *Phyllobacterium trifolii* PETP02	99.39	OQ533475	+++	+
G3	B58	*Agromyces humi* ANK073	98.50	OQ533437	−	+++
	B105	[*Pseudomonas*] *hibiscola* ATCC 19867 *Stenotrophomonas maltophilia* ATCC 19867	99.47	OQ533476	−	+++
	B107	*Staphylococcus capitis* DSM 20329	97.53	OQ533477	+	−
	B108	*Staphylococcus capitis* subsp. *capitis* DSM 20326 *Staphylococcus capitis* JCM 2420	99.30	OQ533478	−	+
G4	B53	*Cellulomonas cellasea* DSM 20118	99.62	OQ533432	++	−
G5	B56	*Alcaligenes faecalis* subsp. *phenolicus* DSM 16503 *Alcaligenes javaensis* JG3	99.59	OQ533435	−	+++
	B57	*Phyllobacterium endophyticum* PEPVI5	91.52	OQ533436	−	++
	B61	*Peribacillus castrilensis* N3 *Peribacillus frigoritolerans* DSM 8801	97.15	OQ533440	−	+++
	B62	*Rhodococcus opacus* DSM 4320/ *Rhodococcus wratislavensis* DSM 44107	98.65	OQ533441	−	+
	B63	*Peribacillus frigotolerans* DSMZ 8801	100	OQ533442	−	++
	B64	*Psychrobacillus lasiicapitis* NEAU-3TGS17	98.55	OQ533443	−	++
	B65	*Psychrobacillus lasiicapitis* NEAU-3TGS17	92.58	OQ533444	−	++
	B66	*Peribacillus frigoritolerans* DSM 8801 *Peribacillus castrilensis* N3	96.69	OQ533445	−	++
	B76a	*Microbacterium foliorum* DSM 12966	98.23	OQ533454	−	++
	B76b	*Micrococcus luteus* NCTC 2665 *Micrococcus yunnanensis* YIM 65004	95.11	OQ606393	−	++
Gx	B80	*Peribacillus frigoritolerans* DSM 8801 *Peribacillus castrilensis* N3	98.42	OQ533458	−	+
P6	B79	*Amycolatopsis jiguanensis* CFHS01580	98.96	OQ533457	−	++
	B83	*Paenibacillus mobilis* S8/ *Paenibacillus tundrae* A10b	98.81	OQ533460	++	+
	B112	*Agromyces terreus* DS-10	99.11	OQ533479	−	−
P7	B37	*Lysinibacillus fusiformis* NBRC 15717	99.04	OQ512987	−	++
	B44	*Lysinibacillus fusiformis* NBRC 15717	99.00	OQ533428	−	+++
	B89	*Promicromonospora xylanilytica* YIM61515	99.27	OQ533466	−	++
	B97	*Micrococcus endophyticus* YIM 56238	97.37	OQ533472	−	+
P8	B60	*Advenella kashmirensis* WT001	98.43	OQ533439	++	+/−
	B67	*Phyllobacterium zundukense* Tri-48 *Phyllobacterium sophorae* CCBAU 03422 *Phyllobacterium loti* S658	96.81	OQ533446	−	+
	B71	*Advenella kashmirensis* WT001	95.01	OQ533449	++	++
	B81	*Peribacillus simplex* NBRC 15720 = DSM 1321 *Peribacillus frigoritolerans* DSM 8801	98.78	OQ533459	−	++
	B85	*Phyllobacterium zundukense* Tri-48 *Phyllobacterium loti* S658 *Phyllobacterium trifolii* PETP02	98.64	OQ533462	++	+
	B92	*Microlunatus parietis* CCM 7636	99.27	OQ533468	−	−
	B102	*Microlunatus nigridraconis* CPCC 203993/ *Microlunatus parietis* CCM 7636	99.31	OQ533474	−	+
P9	B13	*Mesorhizobium atlanticum* CNPSo 3140 *Mesorhizobium comanense* 3P27G6 *Mesorhizobium plurifarium* NBRC 102498	96.81	OQ512977	−	
	B15	*Delftia acidovorans* JCM 5833	99.45	OQ512978	−	+
P10	B04	*Achromobacter pestifer* LMG 3431 *Achromobacter piechaudii* CCUG 724	96.39	OQ512006	−	++
	B06	*Achromobacter xylosoxidans* FDAARGOS_789	99.37	OQ512007	−	+++
	B07	*Bacillus thuringiensis* ATCC 10792	99.25	OQ512008	+	−
	B08	*Brevundimonas diminuta* b71	98.52	OQ512009	−	++
	B09	*Nocardia ninae* DSM 44978	94.95	OQ512010	−	+++
	B10	*Achromobacter insolitus* NCTC13520 *Achromobacter spanius* DSM 23806 *Achromobacter deleyi* LMG 3458	99.40	OQ512011	−	+
	B11	*Delftia acidovorans* JCM 5833	99.09	OQ512975	−	++
	B12 b	*Stenotrophomonas geniculata* ATCC 19374 = JCM 13324	99.40	OQ512976	−	++
	B41	*Bacillus altitudinis* 41KF2b *Bacillus aerophilus* 28K *Bacillus australimaris* MCCC 1A05787 *Bacillus aerius* 24K	98.78	OQ533427	+	+
	B54	*Peribacillus castrilensis* N3 *Peribacillus frigoritolerans* DSM 8801	99.23	OQ533433	−	++
	B55	*Phyllobacterium zundukense* Tri-48 *Phyllobacterium trifolii* PETP02	99.45	OQ533434	+	++
	B70	*Lysinibacillus fusiformis* NBRC 15717	99.05	OQ533448	−	+++
	B74	*Peribacillus frigoritolerans* DSM 8801	98.13	OQ533452	−	++
	B84	*Phyllobacterium zundukense* TRi-48 *Phyllobacterium loti* S658 *Phyllobacterium trifolii* PETP02 *Phyllobacterium bourgognense* STM 201	99.13	OQ533461	++	++
	B86	*Phyllobacterium endophyticum* PEPV15 *Phyllobacterium zundukense* Tri-48 *Phyllobacterium loti* S658	98.05	OQ533463	++	++
PX	B50	*Peribacillus frigoritolerans* DSM 8801 *Peribacillus castrilensis* N3	99.17	OQ533430	−	+
	B72	*Peribacillus frigoritolerans* DSM 8801 *Peribacillus castrilensis* N4	99.43	OQ533450	−	++
	B75	*Peribacillus simplex* NBRC 15720 = DSM 1321 *Peribacillus frigoritolerans* DSM 8801 *Peribacillus castrilensis* N5	99.27	OQ533453	−	++
	B77	*Peribacillus castrilensis* N3/ *Peribacillus frigoritolerans* DSM 8801	99.63	OQ533455	−	+
	B78	*Inquilinus ginsengisoli* Gsoil 080	97.81	OQ533456	+	++
P11	B25	*Myroides odoratus* FDAARGOS_1131	96.76	OQ512979	+	−
	B26	*Providencia rettgeri* FDAARGOS 1450	98.20	OQ512980	+	+
	B27	*Providencia rettgeri* FDAARGOS 1450	98.45	OQ512982	+	+/−
	B28	*Providencia rettgeri* FDAARGOS 1450	98.42	OQ512983	+	+
	B30	*Myroides odoratus* DSM 2801 NBRC 14945	98.51	OQ512984	+	−
	B35	*Lysinibacillus fusiformis* NBRC 15717	98.63	OQ512985	−	++
	B36	*Lysinibacillus fusiformis* NBRC 15717	99.87	OQ512986	−	++
	B38	*Lysinibacillus fusiformis* NBRC 15717	99.91	OQ607474	−	++
	B20, B40	*Peribacillus castrilensis* N3 *Peribacillus frigoritolerans* DSM 8801	99.37	OQ512988	−	++
	B87	*Inquilinus ginsengisoli* Gsoil 080	98.91	OQ533464	++	−
	B88	*Promicromonospora kermanensis* UTMC 533	99.29	OQ533465	−	++
	B90	*Inquilinus ginsegisoli* GSOIL 080	98.98	OQ533467	−	+
	B94	*Microbacterium shaanxiense* CCNWSP60 *Microbacterium arthrosphaerae* CCM 7681 *Microbacterium murale* 01-Gi-001	99.33	OQ533470	−	++
	B95	*Leucobacter aerolatus* CCM 7705	97.37	OQ533471	−	+++
	B96	*Serratia liquefaciens* ATCC 27592	98.61	OQ606395	−	−
	B125	*Achromobacter insolitus* NCTC13520 *Achromobacter spanius* DSM 23806	99.48	OQ533487	−	+++
W-11/b	B116	*Microlunatus parietis* 12-Be-011	98.66	OQ533482	+	+
	B123	*Paenibacillus lautus* NBRC 15380	96.69	OQ533486	+++	−
W-A	B150	*Micromonospora cremea* CR30	98.93	OQ533480	+	+
	B151	*Micromonospora coriariae* DSM 44875 *Micromonospora cremea* CR30	98.81	OQ533481	+	+
W-B	B121	*Nocardioides panzhihuensis* KLBMP 1050	98.28	OQ533485	−	++
	B153	*Leucobacter salsicius* M1-8 *Leucobacter exalbidus* K-540B	99.14	OQ533489	++	−
BALN	B48	*Peribacillus castrilensis* N3 *Peribacillus frigoritolerans* DSM 8801	98.53	OQ533429	−	++
	B52	*Peribacillus frigoritolerans* DSM 8801 *Peribacillus castrilensis* N4	99.72	OQ533431	−	+++
	B69	*Lysinibacillus cavernae* SYSU K30005/ *Lysinibacillus fusiformis* NBRC 15717/ *Lysinibacillus pakistanensis* NCCP-54	97.18	OQ533447	++	−
	B73	*Acinetobacter iwoffii* FDAARGOS 1393	99.47	OQ533451	−	+
	B93	*Prolinoborus fasciculus* CIP 103579	98.30	OQ533469	−	+/−
	B98	*Advenella mimigardefordensis* DPN7/ *Advenella kashmirensis* subsp. *methylica* PK1	97.99	OQ533473	−	++
	B119	*Metabacillus sediminilitoris* DSL-17	99.52	OQ533483	−	++
	B120	*Nocardioides panzhihuaensis* KLBMP1050	98.72	OQ533484	+/−	+
B-14	B128	*Leucobacter aerolatus* CCM 7705/ *Leucobacter salsicius* M1-8	98.74	OQ533488	++	−

Negative result is indicated by −, +/− indicates a weak response, while +, ++, and +++ a positive increasing response.

**Table 2:** Fungal strains identification.

Sampling Point	Isolate	Closest BLASTn Match	% Identity	Accession N
G1	F13	*Penicillium polonicum* KMM4719	99.02	OQ512946
G2	F29	*Aspergillus pseudoglaucus* CBS 126221 *Aspergillus ruber* CBS 126220 *Aspergillus glaucus* CBS 126.55	99.81 99.81 99.81	OQ512947
	F06	*Penicillium oxalicum* DUCC5744	97.82	OQ512948
G3	F19	*Cladosporium neolangeronii* CPC 22267 *Cladosporium psychrotolerans* DTO:305-G3 *Cladosporium langeronii* CPC 22326	100 100 100	OQ512949
G4	F23	*Penicillium chrysogenum* NBPen2012A02	99.33	OQ512950
	F14	*Chrysosporium undulatum* CBS 964.97	99.84	OQ512951
	F22	*Torula hollandica* CBS 220.69	99.18	OQ512952
G5	F28	*Pseudogymnoascus pannorum* CBS 126913	91.60	OQ512953
GX	F38	*Cladosporium halotolerans* CBS 127370 *Cladosporium parahalotolerans* CPC 22373	99.43 99.43	OQ512954
	F39	*Penicillium chrysogenum* SCSGAF0070	99.34	OQ512955
	F31	[*Coniosporium*] MA 4640	97.18	OQ512956
P6	F08	*Penicillium brevicompactum* CBS 287.53	100	OQ512957
P7	F32	*Penicillium dipodomyus* CBS 110412 *Penicillium flavigenum* CBS 419.89 *Penicillium lanosocoeruleum* CBS 334.48	97.90 97.90 97.90	OQ512958
P8	F17	*Cladosporium halotolerans* CBS 114065	99.84	OQ512959
	F04	*Cladosporium parahalotolerans* CPC 22373 *Cladosporium halotolerans* CBS 127370	99.81 99.81	OQ512960
P9	F01	*Malassezia restricta* CBS 7991	98.42	OQ512961
	F02	*Penicillium brevicompactum* CBS 287.53 *Penicillium kongii* AS3.15329	100 100	OQ512962
	F09a	*Cyphellophora olivacea* CCFEE 9916	99.33	OQ512963
P10	F18	*Cladosporium endophyticum* MFLUCC 17-0599	98.46	OQ512964
P11	F05	*Cladosporium herbarum* CBS 128234 *Cladosporium macrocarpum* CBS 12778 *Cladosporium variabile* CBS 121635 *Cladosporium ossifragi* CBS 842.91 *Cladosporium allicinum* CBS 188.54	99.61 99.61 99.61 99.61 99.61	OQ512965
Px	F11	*Penicillium rubens* CBS 132206 *Penicillium chrysogenum* CBS 127368	99.82 99.82	OQ512966
W11	F34	*Penicillium rubens* CBS 132206 *Penicillium chrysogenum* NEF9	97.03 97.03	OQ512967
CV- A	F36	*Penicillium chrysogenum* CBS 127368 *Penicillium rubens* CBS 205.57	99.09 99.09	OQ512968
CV- B	F37	*Penicillium rubens* CBS 132206 *Penicillium oxalicum* DUCC 5744	97.44 97.44	OQ512969
BAL14	F40	*Cladosporium subuliforme* CBS 126500 *C. verrucocladosporioides* CBS 126363 *Cladosporium xylophilum* CBS 125997	99.81 99.81 99.81	OQ512970
BAL15	F41	*Penicillium goetzi* CBS 285.73 *Penicillium rubens* CBS 129667	99.07 99.07	OQ512971

**Table 3:** Total counts of phototrophs and heterotrophs during the multitemporal investigations. To highlight differences occurred over time, the results have been reported as order of magnitude with power of 10 of CFU/cm^2^: +/− indicate positive values below 5; values of power of 10, 100, 1000, and 10,000 are reported as +, ++, +++ and ++++ respectively;); − not found; and / not investigated. Shaded columns are for new data.

Room	Site	2002			2003			July 2019			Nov 2019		
		Photot	Fungi	Bacteria	Photot	Fungi	Bacteria	Photot.	Fungi	Bacteria	Photot.	Fungi	Bacteria
**CG**	G1	−	/	/	−	/	/	−	+++	−	−	+++	−
	G2	−	/	/	−	/	/	−	+++	−	−	+++	−
	G3	/	+	+	/	++	+	/	−	++	/	++	−
	G4	/	+++	−	/	++	++	/	++	−	/	+	+++
	G5	−	+++	++++	−	−	+++	−	+	++	−	+	+++
**NP**	P6	/	−	−	/	+	++	/	−	++	/	+	+
	P7	−	−	+	−	+	+	−	++	+	−	−	++
	P8	/	−	−	/	+	−	/	++	++	/	++	−
	P9	/	+	−	/	+	+	/	+++	+	/	++	−
	P10	++	+	++++	−	−	+++	−	−	++	−	+/−	++
**W**	11	/	+	+	/	+	+	/	−	+	/	−	+
	11/b	/	+	+	/	+	+	/	−	+	/	−	+
	A	−	/	/	−	/	/	+++	+	+	+++	+	+
	B	−	/	/	−	/	/	+++	+	+	+++	+	+
**BAL**	14	/	+++	++	/	+++	+++	+	+	++	+	+	++
	15	/	+++	++	/	+++	+++	+	+	++	+	+	++
	G	+++	/	/	+++	/	/	+	−	+	+	−	+

**Table 4:** Phototrophs identification by morphological and molecular methods, the latter is indicated by asterisk (*). Negative finding has been recorded as – and +/− means not investigated, while +, ++, and +++ a positive increasing finding. Shades indicate new results and samples.

Room	Sample	Deter. Pattern	2002		2003		Deter. Pattern	2019	
**NP**	P10	GBP	*Navicula* sp.	+	−		−	−	
	Px		/		/		LGP	*Scytonema* sp.	+++
**W**	A	GP	−		−		GP	(*) *Pseudostichococcus* cfr. *Monallardoides* *Scytonema* sp.	+++ +/−
	B	YP	−		−		GP	*Chlorella vulgaris*	+++
**BAL**	14		/		/		FGP	(*) *Albertania* cfr. *skiophila* (*) *Coccomyxa* sp.	++ +
	15		/		/		FGP	(*) *Albertania* sp. *Phormidium laminosum*	++ +++
	G	DGP	*Plectonema gracillium* *Desmococcus* sp. *Navicula gallica* Moss *protonemata*	+++ ++ + ++	*Plectonema gracillium* *Desmococcus* sp. *Navicula* gallica *Chlorococcum* sp. *Stichococcus bacillaris* Moss *protonemata*	+++ ++ + + + ++	−	*Desmococcus vulgaris* Fern’ spores	+++ ++
	By		/		/		GOP	Moss gametophyte	+++
	Bx		/		/		DGP	*Chlorococcum vulgaris* *Stichococcus* sp.	+++ +

Quantitative data are based on microscope observations or by the number of extracts from which have been sequenced the species recorded. GBP, grey–brown patina; GP, green patina; YP, yellow patina; DGP, dark-green patina; LGP, light-green patina; FGP, fair-green patina; GOP, green-ochre patina; DGP, dark-green patina.

## Data Availability

All data are available in this article and Supplementary Material.

## Supplementary Materials

The following supporting information can be downloaded at: https://www.mdpi.com/article/10.3390/microorganisms11071770/s1. Figure S1: Bacterial isolates frequency at (A) class, (B) order and (C) family level; Table S1: PCR amplification programs applied for the targeted regions. The last line reports the cycling repeats; Table S2: Environmental conditions recorded during the two sampling sessions.
