# Supplementary material for: The Roman Houses of the Caelian Hill (Rome, Italy): Multitemporal Evaluation of Biodeterioration Patterns
Source: Microorganisms. 2023 Jul 6;11(7):1770. doi: 10.3390/microorganisms11071770 (PMC10384389; doi:10.3390/microorganisms11071770)
Supplement: Supplementary file 1 [file microorganisms-11-01770-s001.zip › microorganisms-2418683-supplementary.pdf]

Supplementary material

The Roman houses of the Caelian Hill (Rome, Italy): Multitemporal evaluation of biodeterioration patterns

Table S1. PCR amplification programs applied for the targeted regions. The last line reports the cycling repeats.

27f<br>1492r	Cya106f<br>Cya781r	ITS5<br>ITS4	rbcLaF<br>rbcLr590	ChloroF<br>ChloroR
95° C x 2'	95° C x 2'	95 °C x 3'	95 °C x 2'	94 °C x 3'
95 °C x 30''	95 °C x 45''	95 °C x 30''	95 °C x 30''	94 °C x 1'
50 °C x 30''	55 °C x 30''	55 °C x 30''	50 °C x 90''	59 °C x 1'
72 °C x 2'	72 °C x 1'	72 °C x 32''	72 °C x 40''	72 °C x 1'
72 °C x 7'	72 °C x 5'	72 °C x 5'	72 °C x 5'	72 °C x 10'
30x	40x	35 x	45x	35 x

Table S2 Environmental conditions recorded during the two sampling sessions.

	outdoor		CG		NP		W		BAL		outdoor	
	°C	RH%	°C	RH%	°C	RH%	°C	RH%	°C	RH%	°C	RH%
July	26.3	61.5	23.3	76.4	22.5	81.3	20.3	88.3	17.7	88.6	31.05	54
Nov	18	72	19.2	68.4	19.4	83.9	19	85	17.5	87.6	16.8	81
hour	10:00 AM		10:15 AM		11:50 AM		1:20 PM		2:35 PM		3:15 PM	
Nov 5th, 2002	15.1	51	18.9	80	19.3	90	20	92	18.3	93		

Outdoor refers to the external conditions recorded in *Clivus Scauri* street just close the entrance door before and after the sampling sessions. CG, Chamber of the Geniuses; NP, Nymphaeum of Proserpine; W, winery; BAL, *Balneum*

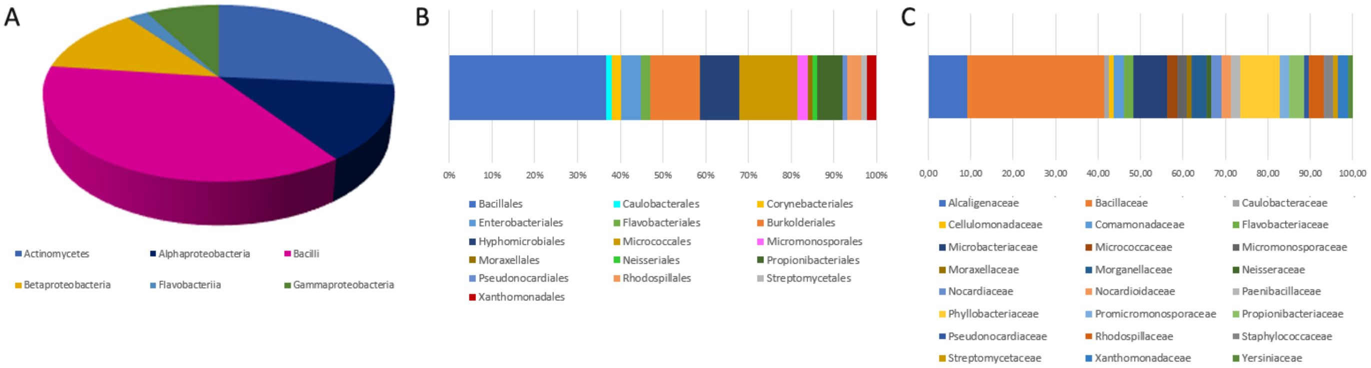

Figure S1. Bacterial isolates frequency at (A) class, (B) order and (C) family level.
